# Human Coinfection with* Borrelia burgdorferi* and* Babesia microti* in the United States

**DOI:** 10.1155/2015/587131

**Published:** 2015-11-30

**Authors:** Kristen L. Knapp, Nancy A. Rice

**Affiliations:** Department of Biology, Western Kentucky University, Bowling Green, KY 42101, USA

## Abstract

*Borrelia burgdorferi*, the causative agent of Lyme disease, and* Babesia microti*, a causative agent of babesiosis, are increasingly implicated in the growing tick-borne disease burden in the northeastern United States. These pathogens are transmitted via the bite of an infected tick vector,* Ixodes scapularis*, which is capable of harboring and inoculating a host with multiple pathogens simultaneously. Clinical presentation of the diseases is heterogeneous and ranges from mild flu-like symptoms to near-fatal cardiac arrhythmias. While the reason for the variability is not known, the possibility exists that concomitant infection with both* B. burgdorferi* and* B. microti* may synergistically increase disease severity. In an effort to clarify the current state of understanding regarding coinfection with* B. burgdorferi* and* B. microti*, in this review, we discuss the geographical distribution and pathogenesis of Lyme disease and babesiosis in the United States, the immunological response of humans to* B. burgdorferi* or* B. microti* infection, the existing knowledge regarding coinfection disease pathology, and critical factors that have led to ambiguity in the literature regarding coinfection, in order to eliminate confusion in future experimental design and investigation.

## 1. Introduction

Tick-borne diseases, which affect both humans and other animals, are on the rise in the United States as once uninhabited wilderness continues to be urbanized promoting increased exposure and transmission to humans. Tick-borne diseases can result from several types of pathogens including bacteria, viruses, and protozoa, and most infections are the consequence of an infected tick bite. Further complicating the diagnosis and treatment of this family of emerging diseases is the fact that ticks can harbor multiple pathogens and coinfect individuals with multiple parasites. Two of the most common tick-borne illnesses in the United States are Lyme disease, a condition caused by the bacterium* Borrelia burgdorferi*, and babesiosis, a disease resulting from infection with* Babesia microti*. These two pathogens occur in overlapping geographic areas in the United States and use the same vector host* Ixodes scapularis*. There is considerable confusion in the literature regarding the effect that coinfection with* B. burgdorferi* and* B. microti* has on disease severity and prognosis. In an effort to clarify what is understood regarding concomitant infection, in this paper, we review the geographical distribution and pathogenesis of Lyme disease and babesiosis in the United States, the immunological response of humans to* B. burgdorferi* or* B. microti* infection, the existing knowledge regarding coinfection disease pathology, and the critical factors that have led to ambiguity in the literature regarding coinfection. A clearer understanding of what is currently known in the literature will serve to help guide future research efforts as well as improve clinical diagnosis and treatment.

## 2. Lyme Disease and Babesiosis

**Figure 1:**
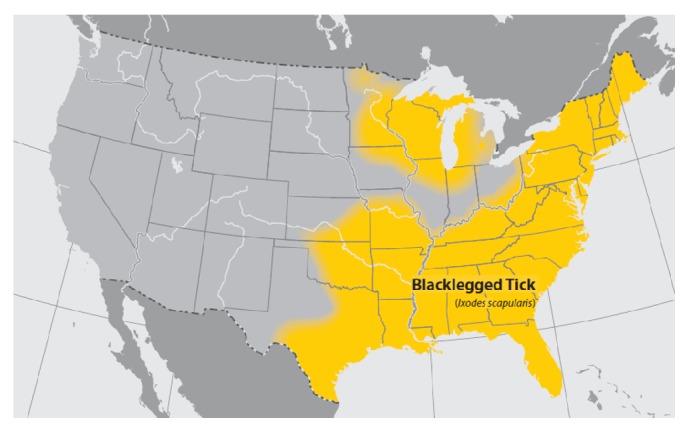
Geographic distribution of* I. scapularis* within the continental United States. Yellow shaded areas represent the distribution of* I. scapularis* in the northeastern and upper midwestern United States [2].

Lyme disease, a condition caused by the bacterium* Borrelia burgdorferi*, is transmitted to humans via the bite of* Ixodes scapularis*, also known by the common names deer tick or blacklegged tick. In 1993,* Ixodes dammini* was proven to be the same species as* I. scapularis*. While the use of the name* I. scapularis* is preferential,* I. dammini* is still occasionally used [1]. For the purpose of this review, the name* I. dammini* will be replaced with* I. scapularis* in order to eliminate confusion. In addition, in the western United States, the Western Blacklegged Tick* Ixodes pacificus* is responsible for the transmission of* B. burgdorferi* to humans [2]. The current geographic distribution of* I. scapularis* in the United States is primarily in the northeastern, southeastern, and midwestern states (Figure 1).

Currently considered the most commonly diagnosed tick-transmitted disease in the United States, Lyme disease affected over 25,000 individuals in 2013 alone according to the Center for Disease Control and Prevention [3]. The overall number of confirmed cases has ranged from a low of 11,700 in 1995 to a high of 29,959 in 2009. Lyme disease is caused by infection with the spirochete bacterium* B. burgdorferi* and initially results in a rash at the site of the infective tick bite followed by flu-like symptoms including fever, chills, fatigue, headache, and general malaise for up to a month. During and after this period, muscular pain, as well as neurological and cardiac pathologies, can occur. In a small percentage of cases, severe complications result such as facial paralysis and dangerous cardiac arrhythmias. Finally, after several months of infection, symptoms similar to those of rheumatoid arthritis begin to occur in joints. A review of the clinical data reveals that infection with* B. burgdorferi* follows often unpredictable disease progression, producing only mild symptoms in some over a long period of time or causing rapid onset of potentially fatal effects in others. Naturally, this observation leads to the question of what factors might influence the variable and unpredictable progression of Lyme disease in the nearly quarter of a million patients infected in the United States every year [4].

In the United States,* I. scapularis* is the most well-known vector of* Babesia microti*, a natural parasite of rodents [5]. Transmitted by the bite of an infected tick and in some rare cases via blood transfusion or congenitally, babesiosis is acquired when sporozoites are introduced into the host's bloodstream by the bite of an infected tick. Following introduction, sporozoites infect erythrocytes, reproduce, and form merozoites that are then capable of erupting from infected erythrocytes and infecting others. The continuation of this cycle yields a large intraerythrocytic population very quickly [5].* B. microti* infection mimics mild malaria with the main symptoms stemming from hemolytic anemia. Indeed,* B. microti* infections are often even diagnosed as malaria due to the similar appearance of infected erythrocytes that can be viewed via a blood smear. While babesiosis can be fatal in immunologically compromised or splenectomized individuals, healthy individuals recover from infection with* B. microti* spontaneously, requiring only temporary treatment of symptoms [5].

**Figure 2:**
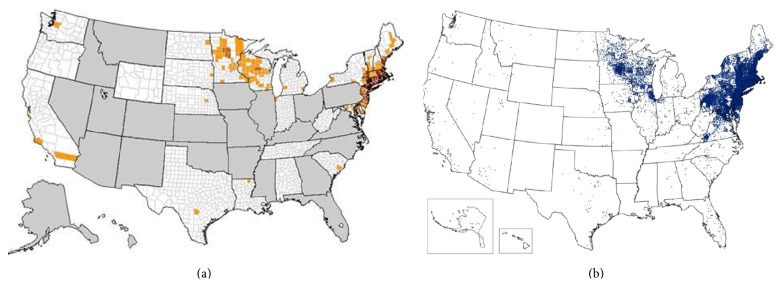
Geographic distribution of babesiosis and Lyme disease in the United States. (a) Reported cases of at least one case of babesiosis in 22 of the 27 different states that conducted surveillance: white = 0 cases; yellow = 1–5 cases; orange = 6–10 cases; brown = 11–20 cases; and dark brown > 20 cases. Babesiosis was not a reportable disease in the gray states, and health departments in those states did not notify CDC of cases [6]. (b) Reported cases of Lyme disease across the United States. Each blue dot represents a confirmed case [7].

Lyme disease and human babesiosis appear most commonly diagnosed in overlapping geographic areas (Figures 2(a) and 2(b)). Babesiosis has been found to be most prevalent in the northeastern United States, as well as the upper midwest, and is diagnosed in particularly high density areas of the northeast, New Jersey, New York, Minnesota, and Wisconsin [6]. Notably, all of these states are also among those that report the largest number of Lyme disease cases each year. In 2011, 96% of Lyme cases were reported from 13 states: Connecticut, Delaware, Maine, Maryland, Massachusetts, Minnesota, New Hampshire, New Jersey, New York, Pennsylvania, Vermont, Virginia, and Wisconsin [7]. The geographic overlap between the occurrence of Lyme disease and babesiosis suggests that the two diseases, both transmitted by the vector* I. scapularis*, may simultaneously infect a population in a geographic area.

Clinical evidence supporting the idea that coinfection may be possible and more common than originally imagined includes, one, the observation that both pathogens are commonly found in rodents captured in endemic areas; two, the notion that patients diagnosed with Lyme disease are often found to also be seropositive for antibabesial antibodies; and three, the fact that* I. scapularis* has been shown to be capable of transmitting both pathogens at once [8–10]. In a study of rodent populations in Prudence and Patience, Rhode Island, the fact that greater than 50% of captured* Peromyscus leucopus* and* Microtus pennsylvanicus* harbored both* B. microti* and* B. burgdorferi* suggests that individual larval* I. scapularis* ingest and transmit both pathogens [11]. Other research has confirmed that rodents and tick vectors are both frequently coinfected. In a study from tick samples captured in New Jersey, of those positive for* B. burgdorferi*,* B. microti*, or human granulocytic ehrlichiosis agent, 20% of ticks were coinfected with at least two pathogens [12]. It is also known that nymphal* I. scapularis* infected with both* B. burgdorferi* and* B. microti* are able to simultaneously transmit both organisms to hamsters [13]. The observation that both pathogens frequently reside together in rodent populations and the tick vector* I. scapularis* certainly raises suspicion that coinfection in human hosts may be possible [14–16]. Multiple studies measuring human antibody titers do provide some evidence that coinfection does occur. These include a recent study that found that up to 66% of residents of Long Island, New York, who were diagnosed with Lyme disease were also seropositive to antibodies against* B. microti* [17, 18]. Conversely, in a study in which patients had first been diagnosed with babesiosis, 54% also possessed IgG and IgM antibodies to* B. burgdorferi* [19]. While the simple presence of antibodies to* B. microti* or* B. burgdorferi* in no way guarantees that both infections were acquired from the same tick, it does raise interesting speculation regarding how temporal variations in the acquisition of babesiosis and Lyme disease, be it from the same tick or within short time frame from different ticks, may affect the clinical progression of coinfection.

Although it is well established that both* B. microti* and* B. burgdorferi* certainly coinfect rodent hosts and tick vectors, that tick vectors are capable of transmitting both pathogens simultaneously, and that a large percentage of patients suffering from Lyme disease or babesiosis have also been exposed to* B. microti* or* B. burgdorferi*, respectively, little research has been amassed regarding the pathophysiological effects of concomitant infection. Citing observations from mice in which babesial infection appears to enhance Lyme disease myocarditis, it has been suggested that coinfection increases the severity of disease and may impair host defense mechanisms [20]. There is some data to support this hypothesis in that patients with coinfections report a longer duration of illness and exacerbated symptoms including myalgia, fatigue, sweats, anorexia, erythema migrans, and conjunctivitis [17, 18]. In one case of coinfection, death as a result of pancarditis even occurred [21]. Other studies, however, report that coinfection does* not* worsen the long term outcome of patients suffering from infection with both pathogens specifically with regard to the prevalence of constitutional, musculoskeletal, or neurological symptoms [22].

Clearly, while it has been established that both* B. microti* and* B. burgdorferi* can coexist in the same organism, infect the same vector, simultaneously infect a mammal host, and cause debilitating symptoms, disagreement is still substantial and research is lacking regarding the synergistic or perhaps only additive effect of concomitant infection. While the debate continues, the incidence of tick-borne infections is quickly on the rise due to a variety of factors such as larger deer populations, increasing tick populations, and increased development of wooded and rural areas bringing humans, deer, and ticks in even closer proximity. As tick-borne infections become more common in the United States and across the globe, the need for research on the clinical manifestation, immunological response, pathophysiological mechanism, and proper treatment of coinfection with tick-borne pathogens is vital.

## 3. Immunological Response to* B. burgdorferi*

The cells of the innate immune system constitute the first line of defense against* B. burgdorferi*. The widely accepted mechanism is that lipid-modified membrane proteins and diacylglycerol-containing glycolipids of the spirochete signal via CD14 and/or Toll-like receptor 2 (TLR2)/TLR1 heterodimers to promote a proinflammatory response during infection [23, 24]. The chemokine receptor CXCR2 also plays a role in the generation of* B. burgdorferi* induced inflammation [25]. Specifically, the lipoproteins and glycolipids of* B. burgdorferi* activate the immune system by binding to TLRs, in particular TLR2, leading to cytokines IL-6, IL-10, IL-12, TNF-*α*, and IL-1*β* being released from innate immune system cells [26]. These cytokines serve as a link between the innate and adaptive immune systems, influencing the response and polarization of the host's cell mediated and humoral immune response against* B. burgdorferi*. Subsequently, as T-helper cells are activated, they differentiate into a combination of Th1, Th2, Th17, or T regulatory cells, resulting in a polarized immune response. Different individuals can mount immune responses with varying polarization, and researchers have speculated that the polarization of the cell mediated immune response may influence the overall outcome of* B. burgdorferi* infection. While not exclusive, the adaptive immune system combats intracellular pathogens via a strong Th1 response, characterized by increased production of IFN-*γ*, while a strong Th2 response, vital for host defense against extracellular pathogens, is characterized by an increase in IL-4 production [26].

In the late 1990s, two studies found that IFN-*γ* predominated, compared to IL-4, during* B. burgdorferi* infection. In one study, researchers noted decreased IL-4 synthesis and increased IFN-*γ* synthesis in patients infected with* B. burgdorferi* compared to a control group [27]. The increase in IFN-*γ* observed in these patients resulted from induced Th1 polarization, contributing to increased pathogenesis of the bacteria, and potential autoimmune reactions. A second report by Ekerfelt and colleagues found similar results in an adult population afflicted with neural* B. burgdorferi* infection. In this population of individuals, suffering from neuroborreliosis, IFN-*γ* production was significantly increased while IL-4 production was unusually low [28]. These two seminal studies suggest that, in adults (particularly those with severe courses of infection), strong Th1 polarization of the cell mediated adaptive immune response is characteristic of* B. burgdorferi* infection.

The observed cytokine response to* B. burgdorferi* also appears to have temporal variability. Individuals with* nonchronic *neuroborreliosis have an initial increase in INF-*γ* followed by an increase in IL-4, corresponding to pathogen clearance, while in individuals who experience* chronic* neuroborreliosis the initial IFN-*γ* response is not followed by IL-4 elevation suggesting a persistent Th1 response [29]. Interestingly, both the genetics and age of the host may influence this temporal immune polarization; children are notably predisposed to generating a highly effective balanced Th1/Th2 response, while adults are more likely to generate primarily a Th1 response [30]. In addition, a strong genetic component involved in the differential immune polarization response to* B. burgdorferi* has been noted in various strains of laboratory mice that exhibit different susceptibilities to* B. burgdorferi* [31].

One of the most significant characteristics of the* B. burgdorferi* spirochete is its ability to avoid immune detection, often for many years, by avoiding the host complement system. The complement system is one of the most versatile parts of the immune system, and its activation leads to phagocytosis of target pathogens or the formation of membrane attack complexes (MACs) [32]. In some cases, host complement regulatory factors are recruited by pathogens in order to protect them from MACs. For example,* B. burgdorferi* recruits host complement proteins factor H (FH) and factor H-like protein-1 (FHL-1) to its own surface, effectively thwarting the host complement attack against the spirochete. Two different borrelial proteins, of the complement regulator-acquiring surface protein (CRASP) family, have been identified as ligands for FH and FHL-1 [33, 34]. Expression of CRASPs directly correlates with serum resistance, in that all serum-resistant isolates express these proteins, whereas all serum-sensitive isolates analyzed to date do not possess proteins with such binding activity [35, 36]. Recently published studies with recombinant outer surface protein OspE suggest that it also functions as a ligand for factor H [37]. Experiments have shown that interference with these surface proteins, particularly OspE, can decrease spirochete survivability making OspE a good therapeutic target [38].

Recently, it was discovered that if* B. burgdorferi* spirochetes were introduced into a host using a syringe versus an infected tick bite, the inflammatory response in the host's skin was altered. When injected via syringe, without associated vector saliva and salivary molecules, the spirochetes elicited an inflammatory reaction characterized by heightened production of TNF-*α* and induction of CRAMP, a mouse cathelicidin (antimicrobial peptide). Alternatively, when mice were inoculated with* B. burgdorferi* via an infected tick bite, the inflammatory response was significantly reduced [39]. Such findings illustrate the importance of natural vector infection and the vital role that vector saliva plays in the establishment of Lyme disease. Initial spirochete multiplication in skin tissue appears to occur prior to dissemination of spirochetes throughout the body, suggesting that if immunity in the skin could be improved or restored via blockade of immunomodulatory and immunosuppressive salivary peptides, disease progression could be delayed or prevented. This idea is further discussed in Natural versus Artificial Inoculation Strategies.

Lastly, the humoral immune response is also vital in protecting the host against deleterious effects of persistent infection. Experiments in a variety of mouse models have shown that both T-cell dependent and T-cell independent mechanisms contribute to activation of B cells and humoral immunity against* B. burgdorferi*. When severe combined immune deficient (SCID) mice were injected with sera from immune competent mice infected with* B. burgdorferi*, the SCID mice were protected from disease even when high doses of spirochetes were used. Conversely, in SCID mice in which infection had already been established, injection with immunocompetent mouse sera resulted in resolution of Lyme arthritis but not carditis, indicating that while a humoral response protects against certain aspects of* B. burgdorferi* infection, cellular and T-cell dependent responses are vital for the complete resolution of infection in all organs [40].

## 4. Immunological Response to* B. microti*

The intraerythrocytic parasite* B. microti* possesses a detailed lifecycle that utilizes several hosts. In nature, both a rodent and tick host are required for survival of the parasite. In the rodent host, most commonly the white-footed mouse* Peromyscus leucopus*, sporozoites are injected from the bite of an infected tick, commonly* I. scapularis*, and infect mouse erythrocytes where they either reproduce asexually or undergo gametogony to produce viable gametes ([5] and the references therein). These gametes are then reintroduced into the definitive host, a tick from the genus* Ixodes*, during a subsequent blood meal. In the definitive host, gametes join to form an ookinete which migrates to the salivary glands and undergoes sporogony producing new sporozoites. In the natural lifecycle of* B. microti*, these sporozoites would once again be injected into a rodent during the process of a tick blood meal. However, if the infected tick instead seeks its blood meal from a human, the sporozoites are introduced into the human host. Humans can also artificially infect others through the process of blood donation and transfusion, as this parasite resides inside erythrocytes for much of its lifecycle. Though not the primary mechanism of infection, babesiosis is the most common blood transfusion-transmitted infection in the United States with 162 cases reported since 1980 [41–43].

The immunological response of the human host against* B. microti* reflects its elaborate lifecycle. Within the human host,* B. microti* infection occurs in three phases: establishment, progression, and resolution [44]. During the establishment phase of infection, sporozoites injected by the bite of an infected tick are free in the plasma. It is during this time that IgG antibodies of a previously exposed host bind to and facilitate the destruction of sporozoites [44]. Once sporozoites penetrate erythrocytes and establish the erythrocytic phase of infection, the innate immune system controls parasite populations during what is referred to as the progression stage. Macrophages producing TNF-*α*, reactive oxygen species, and nitric oxide, as well as natural killer cells producing IFN-*γ*, contribute to the innate immune response, although their mechanism of action is still unknown [44]. Production of IL-12 by macrophages and natural killer cells is also vital for host defense during the progression stage, as mice that lack both macrophages and natural killer cells are unusually susceptible to high levels of parasitemia following* B. microti* infection [45]. The cytokine most vital to the control of parasitemia and resolution of infection may in fact be IFN-*γ*. Not only is IFN-*γ* produced by innate immune cells and effector T cells in both progression and resolution stages of* B. microti* infection, but experimental studies have also found that IFN-*γ* is vital for the generation of protective immunity. In 1999, Igarashi et al. discovered that IFN-*γ* deficient mice were completely incapable of mounting any significant protective immune response against* B. microti*, while blockade of IL-2, IL-4, and TNF-*α* with monoclonal antibodies did not alter the immune response [46]. Finally, the spleen also aids in the process of parasite control as it helps clear damaged and infected erythrocytes through macrophage phagocytosis.

Approximately ten days after* B. microti* infection, parasite numbers generally decrease, and the resolution phase, characterized by activation of CD4^+^, IFN-*γ* producing T cells, begins [44]. The importance of T-helper cells in defense against* B. microti* is well established [47]. The humoral response toward* B. microti* occurs during the resolution stage and results in the production of antibodies specific for surface antigens of merozoites in the plasma. E/S antigens of infected red blood cells help to reduce parasitemia in the blood and protect against future infection. The generation of parasite specific IgG is also essential for the prevention of parasite replication during the resolution stage of* B. microti* infection [48]. Specifically, the humoral response to acute infection is characterized by IgM production, followed by IgG production, and the immunological memory elicited from this response can prevent or reduce the duration and severity of future infections [44].

Based upon the presence of IL-2 and IFN-*γ* throughout* B. microti* infection in mouse models, it is likely that, during the initial stages of infection, establishment, and progression, a Th1 response predominates. IL-2 and IFN-*γ* are present approximately a week after infection and peak around day 12 during the progression stage [49]. Th2 cytokines, IL-4 and IL-10, are elevated starting approximately 2 weeks after infection and peak at three weeks following infection during the resolution stage [49]. Thus, in the early stages of infection, a Th1 response is likely required for the initial control of parasite population growth, while a Th2 response predominates during the resolution stage of infection to clear aging and damaged parasites from the body. Supporting this hypothesis is the observation that the failure to generate and maintain a strong Th1 response during the initials stages of* B. microti* infection results in a drastically increased rate of parasite replication [48]. These results can be extrapolated to human populations as researchers have extensively characterized the human course of babesial infection. Even as early as 1977, it was shown that human subjects experienced a slightly delayed response to* B. microti* with symptoms taking up to two weeks to develop [50].

## 5. Immunological Response to Coinfection with* B. burgdorferi *and* B. microti*

While there is limited research on the host response to concomitant infection with the tick-borne bacterium* B. burgdorferi* and the parasite* B. microti*, it has been suggested that coinfection may result in an altered or suppressed immune response when both pathogens are present. Supporting this, Vinasco et al. observed that, in coinfected BALB/c and C3H mice, the quantity of spleen macrophages is drastically reduced impairing the destruction and clearance of parasitized red blood cells [51]. Thus, such a compromised host immune response could lead to intensified pathogenesis and even the development of chronic infection as suggested in the literature [18, 52].

To examine this further, four key studies, published in an effort to establish definitive conclusions regarding the immunological, pathological, and physiological effects resulting from coinfection with* B. burgdorferi* and* B. microti*, will be reviewed (Table 1). Those factors that have led to uncertainty in the literature regarding coinfection will be discussed, including variations in experimental model (humans versus mice), inoculation strategy, pathogen strain, and quantitative measures.

## 6. Experimental Design Discrepancies

### 6.1. Human Subjects versus Mouse Models

Much of the confusion in the literature regarding coinfection is a direct result of very large differences in experimental design, in particular epidemiological studies from naturally infected humans versus studies in mouse models synthetically inoculated. In general, human studies pose a variety of challenges, one of which is the identification of a large group of patients with acute concomitant infections. While Krause et al. were able to find 26 individuals with evidence of acute coinfection, in the study by Wang et al., only 4 individuals with acute coinfection were identified [18, 22]. These small sample sizes limit the statistical power of the data produced and ultimately result in questionable accuracy for the studies. Furthermore, the wide variety of uncontrollable, confounding variables in human clinical studies, for example, subject variability with regard to medical history, further reduces the accuracy and reliability of results generated from such studies. Human epidemiological analyses constitute two of the four major studies that provide information on concomitant infection with* B. burgdorferi* and* B. microti*. However, the conclusions of these studies are contradictory. Krause et al. found that coinfection with* B. burgdorferi* and* B. microti* results in an increase in pathological severity while Wang et al. determined that coinfection* did not* have an impact on disease or symptom outcomes.

While the aforementioned studies disagree regarding the pathological outcome of coinfection in humans, infections in splenectomized individuals suggest that disease outcomes are indeed synergistic.* B. microti* infection alone is generally a self-limiting disease in healthy, immunocompetent individuals, but splenectomized individuals exhibit characteristically severe pathology with potentially fatal results. A 2008 case study reported that a middle-aged, splenectomized male diagnosed with both* B. microti* and* B. burgdorferi* experienced symptoms of neuroborreliosis only two weeks after an initial diagnosis of babesiosis and failed to respond to multiple antibiotics. The dual infection and associated symptoms were only resolved after complete replacement transfusion with intravenous IgG (IVIG). However, even after five years, the patient still exhibited mild sensory neuropathy in his legs [53].

Although studies using mice models have been able to generate larger sample sizes, control the sample population for previous pathogen exposure and immunological history, regulate timing and mechanism of pathogen exposure, and standardize outcome measurements, these studies, unfortunately, have also produced conflicting results regarding the immunological and pathophysiological effect of coinfection. Some explanations for the continued discrepancy in the literature are that, one, different mouse strains can demonstrate highly varied responses to* B. burgdorferi*; two, the artificial inoculation strategies used eliminated the important variable of the action of tick salivary molecules; and lastly, although mouse models are extremely important in biomedical research, the relevance of nonhuman models in complex immune responses to multiple pathogens is not clear. Two studies using mouse models to investigate whether pathology was exacerbated in the presence of acute coinfection with* B. microti* and* B. burgdorferi* produced conflicting results [54, 55].

Some of the conflict between these studies can be attributed to physiological differences in mouse strains (Table 2). For example, the C3H mouse strain is known to be extremely susceptible to Lyme induced arthritis. In this strain, arthritis severity does not change in coinfected mice versus those singly infected with* B. burgdorferi*, and moreover, splenic weights are also unchanged [55]. However, coinfection in BALB/c mice, a strain much less susceptible to Lyme related arthritis, does present with increased arthritis and decreased IL-10 and IL-13 levels one month after infection, suggesting that additional infection with* B. microti* produces a Th1 inflammatory response responsible for the exacerbated arthritis [54]. The lack of a statistically significant increase in arthritis in C3H mice is likely attributable to the fact that singular infection with* B. burgdorferi* alone already produces an exaggerated Th1 response, possibly due to genetic and immunoinflammatory factors unique to C3H mice; thus, any exacerbation due to coinfection is masked. Interestingly, both mouse strains displayed equal carditis when either singly infected or coinfected. It should be noted that two different substrains of C3H mice were used in these studies, C3H/HeN and C3H/HeJ, which also could lead to variability in experimental results. There is a genetically mediated difference in the two substrains in their response to bacterial endotoxin which is linked to the Toll-like receptor 4 protein; as a result, C3H/HeN mice are endotoxin sensitive, whereas C3H/HeJ mice are endotoxin resistant [56].

### 6.2. Natural versus Artificial Inoculation Strategies

In addition to differences in experimental model, different inoculation strategies have led to disparate results in the literature regarding coinfection with* B. burgdorferi* and* B. microti*. Recently, it was discovered that arthropod saliva contains a variety of proteinaceous factors that enhance a pathogen's ability to establish an initial infection in the host [57]. In many cases, such as that of* Plasmodium*, it appears that arthropod saliva is completely necessary for natural infection to occur. This effect may explain why extremely large doses of* B. burgdorferi* and* B. microti* are necessary to establish infection in the laboratory.

Specifically, salivary proteins of* I. scapularis* were shown to exert unique immunoregulatory effects which aid survival of the pathogens* Rickettsia* and* B. burgdorferi*, not only by creating a more hospitable microenvironment at the injection site, but also by directly assisting in pathogen immune evasion and survival. The salivary factors of* I. scapularis* aid the establishment of* Rickettsia* by inflammatory cytokine suppression and assist* B. burgdorferi* in evading host antibodies by producing Salp15, a salivary protein [58–60]. Another salivary protein, TSLPI (Tick Salivary Lectin Pathway Inhibitor), improves* B. burgdorferi's* transmission through blocking the lectin complement cascade resulting in impaired neutrophil phagocytosis, chemotaxis, and decreased pathogen lysis [61]. The salivary protein Salp25, an antioxidant, reduces ROS concentrations present at the vector-pathogen-host interface via its detoxifying action, thus improving* B. burgdorferi's* chances of survival and successful infection. Salp25, Salp15, TSLPI, and other arthropod salivary proteins, yet to be elucidated, likely play a vital role in the establishment of initial infection within mammalian hosts. As a result of their actions during the host immune system's first exposure to pathogen, these proteins may also cause long term immunomodulatory effects through modifying polarization patterns. Therefore, the absence of arthropod saliva in studies using mouse models likely accounts for some of the incongruous results when compared with clinical case studies.

In addition to the absence of salivary proteins, another problem with artificial inoculation of mouse models is that the injection routes are not anatomically synonymous with that of natural exposure. In most cases, mice are given intraperitoneal, intravenous, or intramuscular injections with the pathogen while natural infection would occur within the dermis or subcutaneous tissue. Altering the initial site of host-pathogen exposure could yield unexpected changes in immune polarization and response since differences in dermal and mucosal immune responses are well known. In both mouse studies discussed in this review, mice were injected intradermally with* B. burgdorferi* spirochetes, thus approximating the natural vector-borne infection pattern; however, in both experiments,* B. microti* parasites were injected intraperitoneally.

### 6.3. Pathogen Strain

Another confounding element in* B. burgdorferi* and* B. microti* coinfection studies is the use of different strains of pathogen. In general, two different strains of* B. microti* have been used in experiments, one isolated from a strain of* P. leucopus*, adapted to growth in laboratory mice and maintained by blood passage in C3H/HeN mice [55], and a second strain, MN1, which was isolated from a human patient, inoculated into golden Syrian hamsters for adaptation, and then cryopreserved rather than maintained by repeated blood passage [62]. The preserved blood was subsequently reconstituted in hamsters for amplification; blood was ultimately collected for studies when 80% parasitemia was reached. It has been hypothesized that the strain adapted from* P. leucopus* may have been attenuated through repeated blood passage, thus producing different results following infection.* B. burgdorferi* strain N40 was used in all studies examined in this review.

### 6.4. Quantitative Measurements

To assess the immunological progression and pathology of coinfection with* B. burgdorferi* and* B. microti* from multiple studies, it is essential that similar measures and outcomes are compared. Several of the most commonly measured immunopathological outcomes of infection are variations in cytokine level, arthritis severity, and peripheral blood pathogen levels. The lack of consistency and standardization in assessment however has generated contradictions in the literature. In Table 3, the methods used in the four major coinfection research studies are compared. Only one study evaluated any change in cytokine level, while all studies performed some variable level of histopathology and serology.

In human epidemiological studies, pathology was determined either by patient self-report or by patient reported symptoms combined with a clinical exam [18, 22]. While the clinical examination used by Wang et al. was standardized based upon the American College of Rheumatology Glossary Joint Exam criteria, this study did not assess all disease parameters, that is, arthritis, neurological changes, and infection status. Overall, Krause et al. reported that in coinfected individuals there was slightly less arthralgia, 27%, compared to either singly infected Lyme disease or babesiosis, 36% and 40%, respectively, although splenomegaly, conjunctivitis, and multiple erythema migrans were all significantly higher in coinfected individuals [18]. This study also confirmed coinfection with* B. burgdorferi* and* B. microti* by the presence of spirochete DNA in blood samples. Spirochete DNA was more frequently detected in coinfected individuals (27%) versus those infected with* B. burgdorferi* alone (6%). Moreover, spirochete DNA was also found for a longer period of time in coinfected individuals versus singly infected ones, mean = 91 days versus 12 days, respectively. Wang et al. only used the presence of antibodies to either* B. burgdorferi* or* B. microti* combined with an acute clinical diagnosis of Lyme disease or babesiosis to confirm coinfection. This approach likely underestimates the number of current or previously coinfected patients, since past coinfection is impossible to confirm with certainty. Furthermore, based upon the serological analyses performed, it is impossible to determine whether actual concurrent coinfection ever existed or whether exposure occurred separately at different time points possibly even years apart. Therefore, much of the data reported by Wang et al. cannot be interpreted regarding the question of increased severity of disease. However, in four patients undoubtedly identified to be acutely coinfected with both* B. burgdorferi* and* B. microti* at the time of the study, disease symptoms indeed lasted longer and were more severe, even resulting in the need for IV medication and prolonged hospitalization in most cases [22].

Unfortunately, mouse studies have not produced any more consistent quantitative outcomes of coinfection [54, 55]. While arthritis in mice is scaled based upon severity by quantifying leukocyte infiltration, there are variations in the scale which are in the range 0–3 versus 0–4. Such variation not only makes objective comparison of results difficult, but also makes replication and verification of results challenging. In addition to different scales of severity being used, the temporal assessment is not consistent across studies. Moro et al. evaluated arthritis severity at 15 and 30 days, while Coleman et al. used a 21-day time point exclusively. Additionally, Moro et al. also quantified IL-4, IL-10, IL-13, and IFN-*γ* cytokine levels throughout the infection process, while Coleman et al. only measured variance in splenic weight, thus making any comparisons with their results more challenging. Clearly, additional investigations using a wide variety of mouse strains are necessary to determine the degree to which coinfection exaggerates disease pathology before a consensus can be reached.

## 7. Conclusions

Overall, it is apparent from this review that there is still much confusion in the literature regarding the pathogenesis and immunological response to coinfection with* B. burgdorferi* and* B. microti*, mostly resulting from experimental design disparities and subject variability. There is undoubtedly great value in the use of naturally infected human subjects in epidemiological studies. While mouse models offer greater control over potentially confounding variables, the mouse immune response, especially in the case of complex coinfection, is likely to be quite different than that of humans making comparisons difficult. However, the conflicting results produced from human epidemiological studies cannot be resolved until outcome measures for arthritis, carditis, neurological manifestations, and immunological determinants are standardized across studies. The human epidemiological studies reviewed in this work were carried out over a decade ago, and significant technological advances have been made since then that can now improve both the identification and recruitment of study participants as well as diagnostic testing and reliability. If future studies standardize assessment parameters, utilize impartial and objective measurements, that is, cytokine quantification, and incorporate larger sample sizes, it is likely that a definitive conclusion can be determined regarding concomitant infection.

Understanding the outcomes of coinfection is increasingly important as Lyme disease and other tick-borne diseases are on the rise. At the time of Krause's seminal study of coinfection with* B. burgdorferi* and* B. microti*, babesiosis appeared to be the primary suspect for coinfection. However, in the years since,* I. scapularis* has been found to transmit not only* B. burgdorferi* and* B. microti*, but also* Anaplasma phagocytophilum*, the bacterium responsible for anaplasmosis, a disease which became nationally reportable in 1999 [58]. It should be noted that, prior to a taxonomic change in 2001 that identified* A. phagocytophilum* as belonging to the genus* Anaplasma*, this bacterium was for many years referred to as* Ehrlichia equi* or* Ehrlichia phagocytophilum*, and the disease caused by this bacterium was frequently termed human granulocytic ehrlichiosis (HGE) [63]. Cases of anaplasmosis have been steadily increasing since 1994 and occur in the same geographic area as Lyme disease and babesiosis.

Coinfection with* B. burgdorferi* and* A. phagocytophilum* has been found to cause increased dispersal of spirochetes in experimentally infected C3H mice, resulting in greater number of spirochetes in ear, heart, and skin tissue [64]. Coinfection results in increased generation of matrix metalloproteinases (MMPs) and increased permeability of microvasculature in the brain increasing the ability of spirochetes to cross the compromised barrier more easily [65]. However, in 2013, Horowitz et al. suggested that coinfection did not increase the severity of disease symptoms [66]. Furthermore, in recent years, concern regarding coinfection with the bacterium* B. miyamotoi*, the causative agent of tick-borne relapsing fever, has further encouraged investigation into the realm of coinfection [67, 68]. Finally, not only has concern regarding concomitant anaplasmosis, Lyme disease, tick-borne relapsing fever, and babesiosis increased in the last decade, but so has the fear of tick-borne viruses such as the Powassan virus or POWV. While the Powassan virus has affected only a small number of individuals in the eastern United States, the effects of this virus are devastating, with over 10% of infected individuals dying due to the characteristic encephalitis and meningitis caused by this pathogen [69].

Overall, the rapid expansion of tick-borne disease in the United States, and the potential for human coinfection with multiple parasites, necessitates that more research be conducted to clarify how coinfection affects disease transmission and progression in order to aid in the accurate diagnosis and treatment of these illnesses.

## Figures and Tables

**Table 1:** Summary of results from four key studies investigating the impact of *B. burgdorferi* and *B. microti* coinfection.

Researchers	Arthritis results	Carditis/other results	Cytokine results	Blood analysis results
Krause et al. (1996) [18]	Increased severity and duration of arthralgia and joint swelling in coinfected individuals^*∗*^	Increased severity and duration of splenomegaly, conjunctivitis, neck stiffness, and erythema migrans in coinfected individuals^*∗*^	N/A	Higher detection of spirochete DNA in peripheral blood in coinfected individuals

Wang et al. (2000) [22]	Equal severity of arthralgia and joint swelling in individuals with evidence of previous babesial infection^∧^	Equal severity of splenomegaly, conjunctivitis, neck stiffness, and erythema migrans in individuals with evidence of babesial infection^∧^	N/A	Higher *Babesia* seropositivity in individuals with evidence of previous babesial infection, 22% versus 7% (control)

Moro et al. (2002) [54]	Increased severity of arthritis in coinfected BALB/c mice 30 days after infection versus single infection; no change in C3H/HeJ mice	Equal severity of carditis in BALB/c and C3H/HeJ coinfected and singly infected mice	Significant decrease in IL-10 and IL-13 in coinfected BALB/c mice 30 days after infection; no change in C3H/HeJ mice	Significant decrease in IgG in coinfected BALB/c mice 15 days after infection which returned to baseline 30 days after infection; no change in C3H/HeJ mice

Coleman et al. (2005) [55]	Equal severity of arthritis in coinfected versus singly infected C3H/HeN mice or BALB/c mice	Equal spleen weights in C3H/HeN or BALB/c coinfected versus singly infected mice	N/A	Equal parasitemia in C3H/HeN or BALB/c coinfected versus singly infected mice; elevated LDH

^*∗*^Symptom severity assessed by patient self-report.

^∧^Symptom severity assessed by clinical exam using criteria set forth by the American College of Rheumatology Glossary Joint Exam.

**Table 2:** Mouse strains.

Strain	Characteristics
BALB/c	Reduced susceptibility to Lyme disease related arthritis Produce IL-4 and develop a Th2 polarized response to *B. burgdorferi* Reduction in IL-4 experimentally demonstrated to increase severity of arthritis Reduced levels of parasitemia in general when infected with *B. microti*

C3H/x^*∗*^	Increased susceptibility to Lyme disease related arthritis Produce IFN-*γ* and develop a Th1 polarized response to *B. burgdorferi* Reduction in IFN-*γ* correlated with reduced arthritis Supplementation with recombinant IL-4 correlated with reduced arthritis

^*∗*^Mouse substrains HeJ and HeN were used in different studies. The difference between the two substrains is that the HeN strain is endotoxin sensitive (normal LPS response) and the HeJ strain is endotoxin resistant.

**Table 3:** Summary of experimental methodology from four key studies investigating the impact of *B. burgdorferi* and *B. microti* coinfection.

Krause et al. (1996) [18]	Human subjects, *n* = 250 *B. burgdorferi* seropositive: *n* = 214 *B. microti* seropositive: *n* = 10 Coinfected: *n* = 26	Epidemiological analyses Clinical evaluation: physical exam and medical history Serological assessment: blood smears, ELISA, western blot, IFA, and PCR Statistical analyses: *χ* ^2^ analysis and Student's *t*-test

Wang et al. (2000) [22]	Human subjects, *n* = 336 Clinical Lyme disease: *n* = 171 Acute concurrent Lyme disease and babesiosis: *n* = 4 *B. burgdorferi* seropositive: *n* = 112 *B. microti* seropositive: *n* = 48 *B. burgdorferi* and *B. microti *seropositive: *n* = 27	Epidemiological analyses Clinical evaluation: physical exam and medical history Serological assessment: western blot and antibody-capture EIA for *B. burgdorfer*i; IFA for *B. microti* Statistical analyses: *χ* ^2^ analysis and Student's *t*-test

Moro et al. (2002) [54]	Mouse model Species: C3H/HeJ and BALB/c	Histopathological analysis for arthritis and carditis Spirochete quantification: competitive PCR Tissue cytokine analysis: ELISA

Coleman et al. (2005) [55]	Mouse model Species: C3H/HeN and BALB/c	Histopathological analysis for arthritis, carditis, and splenomegaly Spirochete quantification: quantitative PCR Serological assessment: ELISA, indirect immunofluorescence assays (IFA), complete blood count, and blood smears
